# The fine-scale architecture of structural variants in 17 mouse genomes

**DOI:** 10.1186/gb-2012-13-3-r18

**Published:** 2012-03-20

**Authors:** Binnaz Yalcin, Kim Wong, Amarjit Bhomra, Martin Goodson, Thomas M Keane, David J Adams, Jonathan Flint

**Affiliations:** 1 The Wellcome Trust Centre for Human Genetics, Roosevelt Drive, Oxford, OX3 7BN, UK; 2 The Center for Integrative Genomics, Department of Medical Genetics, University of Lausanne, Lausanne, Switzerland; 3 The Wellcome Trust Sanger Institute, Hinxton, Cambridge, CB10 1HH, UK

## Abstract

**Background:**

Accurate catalogs of structural variants (SVs) in mammalian genomes are necessary to elucidate the potential mechanisms that drive SV formation and to assess their functional impact. Next generation sequencing methods for SV detection are an advance on array-based methods, but are almost exclusively limited to four basic types: deletions, insertions, inversions and copy number gains.

**Results:**

By visual inspection of 100 Mbp of genome to which next generation sequence data from 17 inbred mouse strains had been aligned, we identify and interpret 21 paired-end mapping patterns, which we validate by PCR. These paired-end mapping patterns reveal a greater diversity and complexity in SVs than previously recognized. In addition, Sanger-based sequence analysis of 4,176 breakpoints at 261 SV sites reveal additional complexity at approximately a quarter of structural variants analyzed. We find micro-deletions and micro-insertions at SV breakpoints, ranging from 1 to 107 bp, and SNPs that extend breakpoint micro-homology and may catalyze SV formation.

**Conclusions:**

An integrative approach using experimental analyses to train computational SV calling is essential for the accurate resolution of the architecture of SVs. We find considerable complexity in SV formation; about a quarter of SVs in the mouse are composed of a complex mixture of deletion, insertion, inversion and copy number gain. Computational methods can be adapted to identify most paired-end mapping patterns.

## Background

The identification of structural variants (SVs) in mammalian genomes [1-4] has important implications for our understanding of genetic diversity, has elucidated the concept of genomic disorders [5,6] and has improved the analysis of genetic association in common and rare diseases [7-12], cancer development [13] and genomic evolution [14,15]. However, the accurate identification of SVs in mammalian genomes remains challenging.

Next generation sequencing provides a novel approach for identifying structural variants [16] and exploits read-pair information [17,18], split reads [19,20], read depth [21] and sequence assembly [22] to localize SVs. Typically, variation in the expected number of reads mapping to the reference sequence is used to identify copy number variants while deviations from the expected distance between reads, and the orientation of reads, is used to infer the presence and type of structural variant at a locus. These methods presuppose that sequencing reads form characteristic patterns for different types of structural variants [23]. For example, when the two sequenced ends of a fragment map back to the reference genome in the correct orientation, but at a distance that is significantly larger than the size of the fragment itself (as inferred from the library insert size distribution), this indicates a deletion.

Algorithms that use whole-genome sequence reads make assumptions about the paired-end mapping (PEM) patterns they will encounter, even though we know that the molecular architecture of structural variants can be remarkably complex [24,25]. For example, deletion and inversion events that appear simple may contain additional sequence at breakpoints and different types of structural variants sometimes occur together, so that, for example, an inversion immediately abuts a deletion [26]. However, current automated methods to identify SVs are unable to differentiate basic patterns (for example, a simple inversion) from more complex ones (for example, an inversion right next to a deletion), resulting in some SVs being incorrectly classified while others are missed altogether.

Past studies have described several forms of complex structural variants, ranging from multiple rearrangements at large genomic loci [27,28], to deletions, inversions, insertions and duplications that fall in close proximity [29]. More recently, a subtle form of complex SVs has been characterized by micro-insertions or micro-deletions at the breakpoints of larger SVs [30]. In our present study, we describe complex SVs as two or more structural variants co-occurring at the same locus, without intervening DNA of normal structure between the variants (SVs are directly adjacent to each other) and without distinction by SV size (complex SVs can be two or more large SVs right next to each other or a small SV right next to a larger SV).

Here we combine visual inspection of PEM data from 17 mouse genomes [31] with experimental validation to resolve the molecular architecture of SVs and to guide genome-wide computational analysis [32]. We provide a comprehensive catalog of 21 PEM patterns derived from simple and complex SVs, and show how these patterns may provide insights into the fine-scale molecular architecture of SV formation.

## Results

### Catalog of paired-end mapping patterns

**Figure 1:**
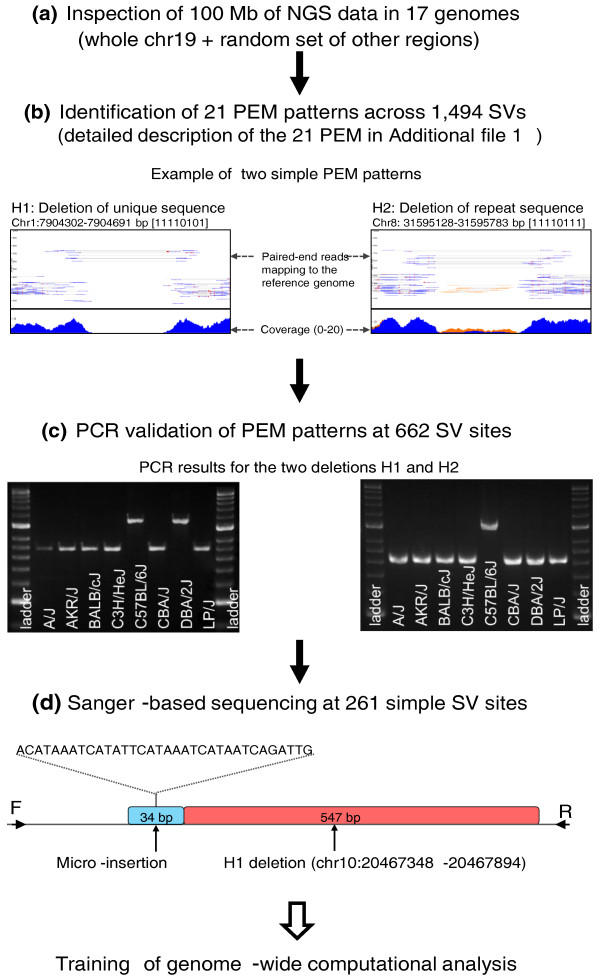
**An overview of the procedure to characterize structural variants**. A flow diagram of the different steps we used to characterize structural variants. **(a) **We first inspected 100 Mbp of next generation sequencing (NGS) data in 17 genomes. We examined chromosome 19 in its entirety and a random set of other chromosomal regions. **(b) **We identified 21 PEM patterns across 1,494 SV sites. We show two examples of PEM patterns, H1 and H2, as visualized using the LookSeq tool [38]. The H1 deletion is on chromosome 1 and has strain distribution pattern 11110101 (1 means presence and 0 absence of the deletion) in the following strain order: A/J, AKR/J, BALB/cJ, C3H/HeJ, C57BL/6J, CBA/J, DBA/2J and LP/J. The H2 deletion is on chromosome 8 and is present in seven strains. **(c) **We randomly selected 662 SV sites for PCR-validation to investigate all PEM patterns and show the results for the two deletion SVs. **(d) **We randomly selected 261 SV sites for analysis of breakpoint sequence features using Sanger-based sequencing technology. We show sequencing data of a simple deletion of type H1 on chromosome 10 (20,467,348-20,467,894). Sequence analysis confirmed the deletion of 547 bp but also revealed an insertion of 34 bp.

We started by generating a set of validated PEM patterns that we could use to guide genome-wide computational analysis. To do this, we visually examined short-read PEM patterns and manually called SVs from 100 Mbp in 17 inbred strains of mice [31,32] (A/J, AKR/J, BALB/cJ, C3H/HeJ, C57BL/6NJ, CBA/J, DBA/2J, LP/J, 129S5SvEv^Brd^, 129P2/OlaHsd, 129S1/SvImJ, NOD/ShiLtJ, NZO/HlLtJ, CAST/EiJ, PWK/PhJ, WSB/EiJ and SPRET/EiJ) that included the whole of mouse chromosome 19 (61 Mbp in size), and a random set of other chromosomal regions. We provide an overview of the procedure to catalog PEM patterns in Figure 1a, b.

**Figure 2:**
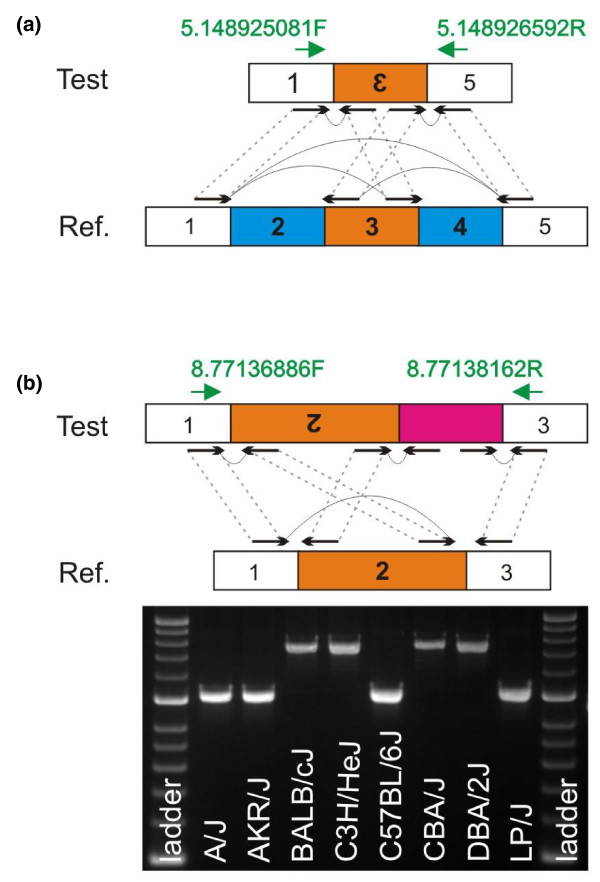
**Novel PEM patterns**. **(a) **PEM pattern of a Del(s)+Inv (H5). We draw paired-end reads (black arrows) and how they map to the reference (Ref.) genome C57BL/6J (dashed grey lines). Blue boxes represent deletions and orange boxes inversions. Green arrows represent primers used for PCR amplification and sequencing reactions. **(b) **PEM pattern of an Inv+Ins (Q8), with PCR data across eight classical strains (A/J, AKR/J, BALB/cJ, C3H/HeJ, C57BL/6J, CBA/J, DBA/2J and LP/J). The pink box represents de novo sequence insertion. The amplicon size for BALB/cJ, C3H/HeJ, CBA/J and DBA/2J is about 500 bp larger than the other strains, indicative of the insertion.

Based on read depth and anomalous PEM, we identified 21 patterns, as described in Table 1 and Additional file 1. We unambiguously classified 11 PEM patterns, referred to as 'H' patterns, for high confidence. While some of the H patterns are typical and have already been described [23], others (H3, H5, H9 and H11) are novel. Figure 2a shows the novel PEM pattern H5, an inversion directly flanked by two deletions. Note that depending on the size of the inversion, the H5 pattern of paired-end reads will differ: for instance, suppose the length of the inversion is small, H5 reads will span both deletions and inversions, giving a PEM pattern of a typical deletion; suppose now the inversion is larger, H5 reads will individually span each deletion, giving a PEM pattern of an inversion.

**Table 1:** The 21 PEM patterns with their corresponding SV type

PEM pattern	Brief description	SV type
H1	Deletion of unique sequence	Simple
H2	Deletion of repeat sequence (for example, LINE, SINE, ERV)	Simple
H3	Deletions separated by small normal copy (Del+Nml+Del)	Simple
H4	Typical inversion	Simple
H5	Inversion co-occurring with deletion(s)	Complex
H6	Insertion of unique sequence (*de novo *sequence)	Simple
H7	Insertion of repeat sequence (for example, LINE, SINE, ERV)	Simple
H8	Tandem duplication	Simple
H9	Inverted tandem duplication	Complex
H10	Dispersed copy number gains	Simple
H11	Deletion or inversion within copy number gain	Complex
Q1	Deletion due to microsatellite size polymorphism	Simple
Q2	Deletion of unique sequence co-occurring with insertion	Complex
Q3	Deletion of repeat sequence co-occurring with insertion	Complex
Q4	Large deletion	Simple
Q5	Linked small gain causing a false deletion	Simple
Q6	False deletion due to retrotransposed pseudogene	False
Q7	Deletion due to VNTR	VNTR
Q8	Inversion co-occurring with insertion	Complex
Q9	Inverted linked small gain causing a false inversion	Simple
Q10	False inversion due to inverted retrotransposed pseudogene	False

ERV, endogenous retrovirus; VNTR, variable number tandem repeat.

Interpretation of the remaining ten PEM patterns was ambiguous. We refer to these as type Q ('Questionable') patterns (Q1 to Q10; Table 1; Additional file 1). With the exception of Q4 (large deletion), the remaining Q patterns have not been described before. Two patterns appeared false positives (Q6 and Q10). Q1 and Q7 were due to variable number tandem repeats. Q5 and Q9 were difficult to interpret: read-pair information suggested a structural variant while read depth did not. Q2, Q3 and Q8 had partial patterns compared to typical SV patterns. For example, Q2 has a read depth of zero flanked by regions of normal read depth but it does not have paired-end reads spanning the length of the variant as does a typical deletion.

We found that partial PEM patterns were caused by the presence of a *de novo *insertion right next to a deletion or inversion. Figure 2b shows an example of an inversion flanked by a *de novo *insertion. A paired-end read spanning the first breakpoint of the inversion is mapped as expected to the reference genome. However, one end of the fragment spanning the second breakpoint of the inversion does not map to the reference genome (because it lies within the inserted sequence), creating a signature of an 'orphaned' read mate.

From the 100 Mbp we visually inspected, we identified a total of 1,494 SVs that matched the 21 PEM patterns (Additional file 1). Because visual identification of H6 and H7 patterns was more difficult than for the other variants, we excluded them from our analysis of chromosome 19 and identified 872 deletions (631 type H and 241 type Q) bigger than 100 bp, 15 inversions (2 type H and 13 type Q) and 3 copy number gains (all type H) (Additional file 2). In addition to the 890 SVs identified on chromosome 19, we found 604 on the other chromosomes. We refer to these lists on chromosome 19 and other chromosomal regions as our gold-standard list of PEM patterns.

Since we examined the whole of chromosome 19 in eight strains, we looked at the distribution of SVs along the chromosome in the context of regional features. To do this, we counted the number of SVs overlapping protein-coding genes, coding exons and repeat regions on chromosome 19 using Ensembl build 65, and we compared this to a null distribution of the expected number of overlaps, obtained by performing a permutation analysis. Across all strains, we found a non-random distribution of SVs along the chromosome (Additional file 3) with enrichment (*P *< 0.01, fold change 2.2) in repeat regions and depletion (*P *< 0.01, fold change 0.25) in coding regions. We found only two SVs on chromosome 19 that affect one, or more, coding exons of genes involved either in immunity or olfaction (Additional file 4).

### Architecture of SVs using PEM inspection

Next we tested the molecular architecture of SVs as inferred by our visual inspection of PEM patterns (Figure 1c). To do this, we applied a primer design strategy depending on type and length of the SV (Additional file 5) and confirmed the underlying molecular structure of all 21 PEM patterns using PCR- and Sanger-based sequencing across 8 (A/J, AKR/J, BALB/cJ, C3H/HeJ, C57BL/6J, CBA/J, DBA/2J and LP/J) of the 17 Mouse Genomes Project strains [31]. These eight strains are the progenitors of the HS (heterogeneous stock) [33], an outbred population we have used to achieve genome-wide high-resolution mapping of multiple phenotypes [34].

We designed 742 pairs of primers (Additional file 6) and successfully amplified 662 SV sites (Additional file 7). It should be noted that we excluded failed designs (due to the presence of SNP(s) in the primer sequences) and designed 80 additional primer pairs to amplify sites when one primer pair yielded no information or only part of the answer - for instance, for a deletion adjacent to an insertion predicted by visual inspection of the PEM. With the exception of insertions (excluded from our chromosome 19 data set), SV sites we analyzed were representative of the overall distribution of PEM categories (Additional file 8).

**Figure 3:**
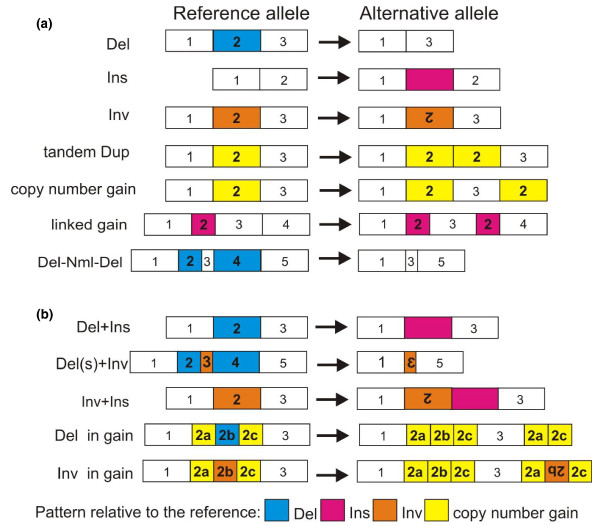
**Architecture of structural variants**. **(a) **Simple SVs: deletion (Del), insertion (Ins), inversion (Inv), tandem duplication (tandem Dup) and other types of copy number gains. Linked gain is a small copy number gain at close proximity to its copy. Inverted linked gain (not drawn) is similar to a linked gain but the copy is inverted. Del+Nml+Del is two deletions separated by a normal copy of small size. **(b) **Complex SVs: deletion co-occurring with insertion (Del+Ins), inversion with flanking deletions (Del(s)+Inv), inversion with insertion (Inv+Ins), deletion within a copy number gain (Del in gain) and inversion within a copy number gain (Inv in gain).

We defined simple SVs as those whose structural interpretation is straightforward and consists of one SV type: insertions, deletions and inversions (Figure 3a). We also identify another type of insertion, a copy number gain, as consisting of non-repetitive DNA that is present in multiple copies relative to the reference genome. When this sequence occurs immediately adjacent to its original, it is annotated as tandem duplication; when it is small and close to another copy, it is annotated as a linked gain.

In contrast to previous SV studies that use the number of breakpoints that fall in close proximity, our definition of complex SVs is based on the mixture of SV types (of small or large size) that directly abut each other, with no intervening DNA, since these might be the progeny of a single process (marked as Del+Ins, Del(s)+Inv and Inv+Ins in Figure 3b). We also separately identify an SV within a copy number gain (termed 'Del in gain' and 'Inv in gain' in Figure 3b) since the probability of coincidence is less than one event per genome.

Our categorization of predicted SV structures, based on manual inspection of PEM patterns, resulted in the highly confident identification of a structural variant for 18 of the 21 patterns that we examined by PCR: 12 were indicative of a simple SV and 6 of a complex SV (Table 1). Two patterns did not represent structural variants (Q6 and Q10), but were due to the presence of a retrotransposed pseudogene, which caused false SV patterns. SVs of type Q7 (55 cases) were due to variable number tandem repeats, for which we could not predict the number of repeats or molecular structure (Additional file 8).

We estimated the relative proportions of simple and complex SVs by manual inspection of PEM patterns on chromosome 19. Assuming an equal number of deletions and insertions on chromosome 19, then about 88% of SVs are composed of one SV, 2.5% of two adjacent SVs at the same locus and 9.5% are variable number tandem repeats (Additional file 8). Note that we have not recorded SVs on chromosome 19 where three (or possibly more) different types of SVs co-occurred (for example, a deletion right next to an inversion and insertion), although about a dozen rearrangements had three different types of SVs based on their PEM patterns. Consequently, our estimate of the number of complex SVs on chromosome 19 based on PEM inspection is likely to be conservative.

### Fine-scale architecture of simple SVs

To gain insights into the fine scale architecture of simple SVs as inferred by our visual inspection of PEM patterns, we randomly selected 261 simple SV sites and analyzed their breakpoints (Figure 1d; Additional file 8). Using the rat as an outgroup species, we inferred SV ancestry (as described in [32]), and classified SVs into two groups (ancestral insertion or deletion). We found 105 ancestral insertions and 142 ancestral deletions; the remaining 14 were either inversions (*n *= 6) or tandem duplications (*n *= 8). We sequenced a total of 4,176 breakpoints at 261 selected SV regions in 8 strains using PCR-based Sanger sequencing (Additional file 9).

We found additional complexities at breakpoints of 62 SVs (24%): small additional (micro-insertions) and small missing (micro-deletions) sequences (Table 2). Figure 1d shows an example of a simple H1 deletion; sequence analysis at the nucleotide level confirmed the deletion of 547 bp but also revealed an insertion of 34 bp.

**Table 2:** Sanger-based sequence analysis at 4,176 breakpoints

Sequence features at breakpoint	Ancestral insertion	Ancestral deletion	Inversion	Gain
Micro-deletion				
None	84.8%			
1-34 bp	14.3%		66.7%	
>200 bp	1.0%			
				
Micro-insertion				
None		73.2%		87.5%
1-10 bp		19.7%		12.5%
11-50 bp		5.6%		
>51 bp		1.4%		
				
Both micro-deletion and -insertion				
1-10 bp				
11-50 bp			16.7%	
>51 bp			16.7%	
				
**Total simple SVs analyzed = 261**	**105**	**142**	**6**	**8**

We determined the extent and content of micro-insertions and micro-deletions and identified three patterns of SV breakpoints (classified in Table 2). The first pattern is characterized by micro-deletions at SV breakpoints. At 15% of ancestral insertions there were missing nucleotides at the breakpoints, ranging from 1 to 289 bp.

The second pattern includes SVs that have sequence inserted at their breakpoints; 27% of ancestral deletions showed a micro-insertion, with size ranging from 1 to 107 bp. We report the origin of the sequence involved in micro-insertions in Additional file 9. There were three cases: (i) intra- or (ii) inter-chromosomal copy number gain of small size, or (iii) insertion of retrotransposons.

The third pattern of SV breakpoints is characterized by simple SVs with both occurrence of micro-deletion and micro-insertion. One third of inversions had this pattern at their breakpoint. Size of SVs was not correlated to one particular pattern, nor to micro-deletion/micro-insertion length and type.

Our analysis of breakpoint sequence features in multiple strains also allowed us to look for a relationship between sequence variants and SV formation. In particular, we addressed the question as to whether sequence variants at breakpoints were associated with SV formation.

**Figure 4:**
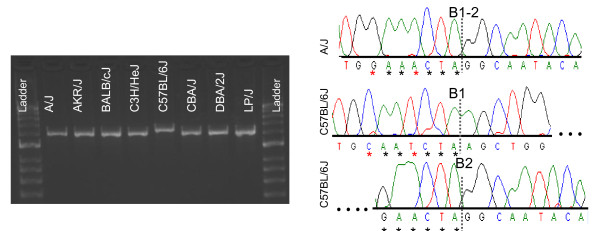
**Relationship between SNP and SV formation**. Two SNPs lying on the 6 bp micro-homology of an ancestral deletion of 64 bp (chr12:27,040,459-27,040,522) correlated with the presence of the SV. Sequencing traces are shown for a test strain (A/J) and the reference strain (C57BL/6J). Note that all other test strain traces are identical to the one shown here. The black asterisks indicate the micro-homology of 6 bp (GAACTA). The presence of two SNPs (C->G and T->A, shown using red asterisks) in all test strains (here only shown in A/J) is associated with the presence of the structural variant.

In all cases, the presence of SNPs in the micro-homology region (short length of identical sequence at an ancestral deletion's start and end points) was correlated with the presence of the SV (Figure 4). The SNP elongates the micro-homology, or, alternatively, the micro-homology reflects a hyper-mutable state associated with break-induced replication around the SV [35]. However, this phenomenon is rare: we only observed five (4.5%) cases amongst our manually curated ancestral deletions (Additional file 9) where a SNP and SV formation co-segregate. We found a similar relationship between a SNP formed at the target site duplication and the presence of an ancestral insertion. Fifteen ancestral insertions (16%) had SNPs or short indels within their target site duplication, coincident with the insertion (Additional file 9).

## Discussion

Our findings are important in two ways. First, we show that an integrative approach using experimental analyses to train computational SV calling is essential for the accurate characterization of SV architecture. Second, we find a considerable complexity in SV formation; about a quarter of SVs in the mouse are composed of a complex mixture of deletion, insertion, inversion and copy number gain.

In contrast to studies that start by identifying SVs using automated genome-wide methods, followed by experimental validation, we started by experimentally determining a set of SVs and then applied this information to interpret whole-genome automated SV detection [32]. Laboratory-based efforts proved essential for two main reasons. First, they allowed the correct interpretation of the PEM patterns. Without knowing how to interpret the underlying molecular structure of each PEM, some patterns would be missed or classified incorrectly by computational methods alone. Second, our laboratory efforts allowed the recognition of a diversity of PEM patterns. Otherwise we would not be able to distinguish between simple and complex SVs.

Finer-scale breakpoint sequence analysis reveals that 24% of simple SVs have smaller rearrangements at the nucleotide level (micro-insertions or micro-deletions at the breakpoint of a larger SV). This raises questions about the likely mechanisms of SV formation.

We know that retrotransposition is the commonest mechanism of SV formation in the mouse [32]. We also know that retrotransposons (LINEs, SINEs and long terminal repeats) are typically characterized by flanking target site duplications and a poly(A) tail or poly(T) head. However, we observed that 15% of retrotransposon SVs do not have target site duplications and truncated or absent poly(A) tails or poly(T) heads (Additional file 9). Moran and colleagues [36] observed a similar phenomenon in the human genome and suggested that retrotransposons, such as LINE-1 elements, integrate into DNA lesions, resulting in retrotransposon-mediated DNA repair. We suggest that about 15% of retrotransposon SVs in the mouse genome formed through a similar mechanism involving DNA repair.

It is reasonable to assume that the complexities (micro-insertions and micro-deletions) we see at the breakpoints of ancestral deletions, inversions and gains (we call these 'complex' non-retrotransposon SVs) (Table 2) will correlate with a complex mechanism of formation. A DNA replication fork stalling and template switching/microhomology-mediated break-induced replication (FoSTeS/MMBIR) mechanism has been proposed to generate such complex SVs in the human genome [37]. In addition, about half of our complex non-retrotransposon SVs have microhomology (short sequence of identical bases) ranging from 3 to 25 bp (Additional file 9), compatible with a microhomology-mediated break-induced replication process. It could be that the complex non-retrotransposon SVs are also the progeny of mutational processes during DNA replication.

Interestingly, our estimate that 24% of SVs have micro-structures at their breakpoint is the same as that reported by Eichler and colleagues [30] in a study of human structural variation. Another sequencing-based study of SVs in two mouse strains (DBA/2J and C57BL/6J) examined 3,316 breakpoints and reported that 16% of non-transposon structural variants are complex, as defined by multiple breakpoints mapped to within 1 kbp of each other [29]. However, we were not able to directly compare these results to ours since we have not used the same classification criteria (we used a classification based on SVs being right next to each other, whereas Hall and colleagues' [29] was based on SVs being at close proximity).

Ideally, sequencing longer reads would typically be required to resolve the complex architecture of structural variants we report in this study, something that goes beyond the current generation sequencing platforms. Our findings offer an intermediate solution between next generation sequencing analysis and complete *de novo *assembly of genomes.

## Materials and methods

### Visual identification of PEM patterns

We visually inspected short-read sequencing data using LookSeq [38] and manually detected PEM patterns across mouse chromosome 19 in its entirety and a random set of other chromosomal regions, accounting for 100 Mbp of total genomic regions. We analyzed molecular architecture of these PEM patterns at nucleotide-level resolution using PCR and Sanger-based sequencing.

### Experimental validation of SV architecture

Primers were designed using Primer3 [39] and purchased from MWG (Ebersberg, Germany). Primer design strategy was dependent on the type and size of the structural variant (Additional file 5). Three independent PCR reactions were carried out with Hotstar Taq obtained from Qiagen (Hilden, Germany). Reactions were performed as previously described [40]. LongRange PCR kit (Qiagen) was used for genomic regions > 2 kbp. PCR gel images were then assessed for quality of primer design and performance of PCR reaction. Representative PCR examples are given in Additional file 1. We provide interested readers with PCR results for each SV site (Additional file 7). Complex SV sites were subject to several rounds of primer design and PCR. PCR products were then purified in a 96-well Millipore purification plate, resuspended in 30 μl of H_2_O and sequenced as previously described [40]. All sequencing reactions were run out on an ABI3700 sequencer and assembled by using PHRED/PHRAP [41].

### Assembly of Sanger-based sequencing data

Consed was used for visualization and editing of the assembly [42]. Strains with and without the SV were aligned into one contig. Breakpoint analysis was mostly based on visual inspection of the alignment and BLAT search. The first breakpoint was identified when the strains with and without the SV stopped aligning and the second breakpoint when they started aligning again. Micro-insertion at the breakpoint was defined as any additional sequence relative to the reference genome (MGSCv37) in the strains with a deletion variant. Micro-deletion at the breakpoint was defined as any missing sequence relative to the reference genome (MGSCv37) in the strains with insertion variant. We also recorded the longest stretch of sequence identity at both breakpoints, which we defined as sequence micro-homology. When micro-homology occurred, we recorded the SV so that start and end coordinates were the smallest. For blunt ended SV, one set of start and end coordinates was recorded. BLAT was used to get the exact start and end coordinates of the SV.

### Genome-wide detection of structural variants

Genome-wide structural variants were detected using four methods: split-read mapping (Pindel) [20], mate-pair analysis (BreakDancer) [18], single-end cluster analysis (SECluster and RetroSeq, unpublished), and read-depth (CND) [21]. Details of the complete pipeline, SVMerge, is described elsewhere [43]. We used in-house Perl scripts to detect genome-wide complex structural variants [32].

#### Data

Data sets described in this study are available under study accession number 'estd185' from the Database of Genomic Variants Archive (DGVa) [44]. Our previous genome-wide data of structural variants [32] are also available from DGVa under accession number 'estd118'.

## Abbreviations

bp: base pair; Del: deletion; Dup: duplication; kbp: kilobase pair; Ins: insertion; Inv: inversion; LINE: long interspersed nuclear element; Mbp: megabase pair; Nml: normal; PCR: polymerase chain reaction; SINE: short interspersed nuclear element; PEM: paired-end mapping; SNP: single nucleotide polymorphism; SV: structural variant.

## Competing interests

The authors declare that they have no competing interests.

## Authors' contributions

DJA and JF directed the research. KW and TMK performed the genome-wide SV discovery. KW wrote computational methods to detect complex SVs. AB and BY analyzed Sanger-based sequencing data. BY cataloged PEM patterns, determined molecular architecture of SVs and led experimental analyses. MG carried out additional analyses. BY and JF wrote the paper. All authors read and approved the final manuscript.

## Supplementary Material

Additional file 1 **21 PEM patterns**. We found 11 'high-confidence' patterns and 10 'questionable' patterns. For each PEM, we provide PEM details, illustration using LookSeq [38] and PCR results. We show paired-end reads (black arrows) and how they map to the reference genome (dashed grey lines). Green arrows represent primer pairs. PCR was carried out across the founder strains of the HS [33]. We used HyperladderII as size marker. Click here for file

Additional file 2 **Chromosome 19 gold-standard data set**. Columns 1 to 3: chromosome, approximate SV start and end coordinates (bp). Column 4: SV length (bp). Column 5: PEM pattern (Table 1; Additional file 1). Columns 6 to13: strain distribution pattern (SDP) across eight classical strains (1 = SV present; 0 = SV absent). Column 14: has SV been PCRed (1 = yes). Click here for file

Additional file 3 **Distribution of manual SV calls along chromosome 19**. The top horizontal tracks show the chromosomal distribution of manually identified structural variants (deletions, inversions and duplications) for specific mouse strains (A/J, AKR/J, BALB/cJ, C3H/HeJ, CBA/J, DBA/2J and LP/J). The bottom two tracks represent genes (Ensembl 65) and gaps on chromosome 19. Click here for file

Additional file 4 **Chromosome 19 manual SV calls that affect coding regions**. Column 1: chromosome. Columns 2 and 3: SV start and stop coordinates (bp). Column 4: SV event. Column 5: affected gene (a plus sign indicates that the gene is affected in its entirety). Column 6: description of the gene. Click here for file

Additional file 5 **Primer design strategy**. We applied a primer design strategy depending on type and length of the SV. Forward primer is in green and reverse primer in red. SV sites were repeat masked prior to primer design, using RepeatMasker [45]. Breakpoints were initially predicted using LookSeq [38]. Primer design is illustrated for: **(a) **tandem duplication, **(b) **insertion, **(c) **deletion and **(d) **inversion. Click here for file

Additional file 6 **Primers**. For each primer pair (PP), we provide a primer pair identification, name and sequence of forward and reverse primers. Click here for file

Additional file 7 **PCR data in eight classical strains**. Column 1: chromosome. Columns 2 and 3: SV start and end coordinates (bp). Column 4: SV length. Column 5: PEM pattern (Table 1; Additional file 1). Columns 6 to 13: 1 = presence or 0 = absence of the SV (2, 3 and 4 indicate multi-allelic SVs). Column 14: has the site been (= 1) or not (= 0) resolved at nucleotide level resolution (when column 14 = 1, columns 2 and 3 refer to the exact coordinates, otherwise they are estimates). Column 15: primer coverage (number of primer pairs designed per unique SV site). Column 16: primer pair used to amplify the SV region. Click here for file

Additional file 8 **Summary data of PCR and Sanger-based sequencing for each of the 21 PEM patterns**. Column 1: PEM pattern (Table 1; Additional file 1). Column 2: number of unique SV sites PCRed. Column 3: chromosome 19 data (some cells are marked NA (not applicable) because we have not systematically inspected H6 and H7 patterns). Column 4: predicted SV. Column 5: PCR validated SV. Column 6: number of SVs sequenced at nucleotide level. Column 7: type of SV as simple, complex, false or variable number tandem repeat. Click here for file

Additional file 9 **The 261 simple SV sites resolved at nucleotide level resolution using Sanger-based sequencing**. Column 1: primer name. Columns 2 to 5: exact SV position. Column 6: PEM pattern. Column 7: length of any micro-deletion or micro-insertion at the SV breakpoint (bp). Columns 8 to 15: strain distribution pattern SDP. Columns 16 and 17: is there a SNP within the micro-homology (MH) or target site duplication (TSD) (0 = no, 1 = yes; NA, not applicable). Column 18: MH length (bp). Column 19: MH type. Column 20: TSD length. Column 21: origin of the inserted sequence. Click here for file

## References

[B1] Conrad DF Pinto D Redon R Feuk L Gokcumen O Zhang Y Aerts J Andrews TD Barnes C Campbell P Fitzgerald T Hu M Ihm CH Kristiansson K Macarthur DG Macdonald JR Onyiah I Pang AW Robson S Stirrups K Valsesia A Walter K Wei J Tyler-Smith C Carter NP Lee C Scherer SW Hurles ME Origins and functional impact of copy number variation in the human genome. Nature 2010 464 704 712 10.1038/nature08516 19812545 PMC3330748

[B2] Kidd JM Cooper GM Donahue WF Hayden HS Sampas N Graves T Hansen N Teague B Alkan C Antonacci F Haugen E Zerr T Yamada NA Tsang P Newman TL Tuzun E Cheng Z Ebling HM Tusneem N David R Gillett W Phelps KA Weaver M Saranga D Brand A Tao W Gustafson E McKernan K Chen L Malig M Mapping and sequencing of structural variation from eight human genomes. Nature 2008 453 56 64 10.1038/nature06862 18451855 PMC2424287

[B3] Mills RE Walter K Stewart C Handsaker RE Chen K Alkan C Abyzov A Yoon SC Ye K Cheetham RK Chinwalla A Conrad DF Fu Y Grubert F Hajirasouliha I Hormozdiari F Iakoucheva LM Iqbal Z Kang S Kidd JM Konkel MK Korn J Khurana E Kural D Lam HY Leng J Li R Li Y Lin CY Luo R Mapping copy number variation by population-scale genome sequencing. Nature 2011 470 59 65 10.1038/nature09708 21293372 PMC3077050

[B4] Redon R Ishikawa S Fitch KR Feuk L Perry GH Andrews TD Fiegler H Shapero MH Carson AR Chen W Cho EK Dallaire S Freeman JL Gonzalez JR Gratacos M Huang J Kalaitzopoulos D Komura D MacDonald JR Marshall CR Mei R Montgomery L Nishimura K Okamura K Shen F Somerville MJ Tchinda J Valsesia A Woodwark C Yang F Global variation in copy number in the human genome. Nature 2006 444 444 454 10.1038/nature05329 17122850 PMC2669898

[B5] Lupski JR Genomic disorders: structural features of the genome can lead to DNA rearrangements and human disease traits. Trends Genet 1998 14 417 422 10.1016/S0168-9525(98)01555-8 9820031

[B6] Lupski JR Genomic disorders ten years on. Genome Med 2009 1 42 10.1186/gm42 19439022 PMC2684663

[B7] McCarroll SA Extending genome-wide association studies to copy-number variation. Hum Mol Genet 2008 17 R135 142 10.1093/hmg/ddn282 18852202

[B8] McCarroll SA Altshuler DM Copy-number variation and association studies of human disease. Nat Genet 2007 39 S37 42 10.1038/ng2080 17597780

[B9] Pinto D Pagnamenta AT Klei L Anney R Merico D Regan R Conroy J Magalhaes TR Correia C Abrahams BS Almeida J Bacchelli E Bader GD Bailey AJ Baird G Battaglia A Berney T Bolshakova N Bolte S Bolton PF Bourgeron T Brennan S Brian J Bryson SE Carson AR Casallo G Casey J Chung BH Cochrane L Corsello C Functional impact of global rare copy number variation in autism spectrum disorders. Nature 2010 466 368 372 10.1038/nature09146 20531469 PMC3021798

[B10] Bochukova EG Huang N Keogh J Henning E Purmann C Blaszczyk K Saeed S Hamilton-Shield J Clayton-Smith J O'Rahilly S Hurles ME Farooqi IS Large, rare chromosomal deletions associated with severe early-onset obesity. Nature 2010 463 666 670 10.1038/nature08689 19966786 PMC3108883

[B11] Jarick I Vogel CI Scherag S Schafer H Hebebrand J Hinney A Scherag A Novel common copy number variation for early onset extreme obesity on chromosome 11q11 identified by a genome-wide analysis. Hum Mol Genet 2011 20 840 852 10.1093/hmg/ddq518 21131291 PMC3024044

[B12] Xu B Woodroffe A Rodriguez-Murillo L Roos JL van Rensburg EJ Abecasis GR Gogos JA Karayiorgou M Elucidating the genetic architecture of familial schizophrenia using rare copy number variant and linkage scans. Proc Natl Acad Sci USA 2009 106 16746 16751 10.1073/pnas.0908584106 19805367 PMC2757863

[B13] Diskin SJ Hou C Glessner JT Attiyeh EF Laudenslager M Bosse K Cole K Mosse YP Wood A Lynch JE Pecor K Diamond M Winter C Wang K Kim C Geiger EA McGrady PW Blakemore AI London WB Shaikh TH Bradfield J Grant SF Li H Devoto M Rappaport ER Hakonarson H Maris JM Copy number variation at 1q21.1 associated with neuroblastoma. Nature 2009 459 987 991 10.1038/nature08035 19536264 PMC2755253

[B14] Sudmant PH Kitzman JO Antonacci F Alkan C Malig M Tsalenko A Sampas N Bruhn L Shendure J Eichler EE Diversity of human copy number variation and multicopy genes. Science 2010 330 641 646 10.1126/science.1197005 21030649 PMC3020103

[B15] Itsara A Wu H Smith JD Nickerson DA Romieu I London SJ Eichler EE De novo rates and selection of large copy number variation. Genome Res 2010 20 1469 1481 10.1101/gr.107680.110 20841430 PMC2963811

[B16] Alkan C Coe BP Eichler EE Genome structural variation discovery and genotyping. Nat Rev Genet 2011 12 363 376 10.1038/nrg2958 21358748 PMC4108431

[B17] Korbel JO Urban AE Affourtit JP Godwin B Grubert F Simons JF Kim PM Palejev D Carriero NJ Du L Taillon BE Chen Z Tanzer A Saunders AC Chi J Yang F Carter NP Hurles ME Weissman SM Harkins TT Gerstein MB Egholm M Snyder M Paired-end mapping reveals extensive structural variation in the human genome. Science 2007 318 420 426 10.1126/science.1149504 17901297 PMC2674581

[B18] Chen K Wallis JW McLellan MD Larson DE Kalicki JM Pohl CS McGrath SD Wendl MC Zhang Q Locke DP Shi X Fulton RS Ley TJ Wilson RK Ding L Mardis ER BreakDancer: an algorithm for high-resolution mapping of genomic structural variation. Nat Methods 2009 6 677 681 10.1038/nmeth.1363 19668202 PMC3661775

[B19] Albers CA Lunter G MacArthur DG McVean G Ouwehand WH Durbin R Dindel: accurate indel calls from short-read data. Genome Res 2011 21 961 973 10.1101/gr.112326.110 20980555 PMC3106329

[B20] Ye K Schulz MH Long Q Apweiler R Ning Z Pindel: a pattern growth approach to detect break points of large deletions and medium sized insertions from paired-end short reads. Bioinformatics 2009 25 2865 2871 10.1093/bioinformatics/btp394 19561018 PMC2781750

[B21] Simpson JT McIntyre RE Adams DJ Durbin R Copy number variant detection in inbred strains from short read sequence data. Bioinformatics 2010 26 565 567 10.1093/bioinformatics/btp693 20022973 PMC2820678

[B22] She X Jiang Z Clark RA Liu G Cheng Z Tuzun E Church DM Sutton G Halpern AL Eichler EE Shotgun sequence assembly and recent segmental duplications within the human genome. Nature 2004 431 927 930 10.1038/nature03062 15496912

[B23] Medvedev P Stanciu M Brudno M Computational methods for discovering structural variation with next-generation sequencing. Nat Methods 2009 6 S13 20 10.1038/nmeth.1374 19844226

[B24] Liu P Erez A Nagamani SC Dhar SU Kolodziejska KE Dharmadhikari AV Cooper ML Wiszniewska J Zhang F Withers MA Bacino CA Campos-Acevedo LD Delgado MR Freedenberg D Garnica A Grebe TA Hernandez-Almaguer D Immken L Lalani SR McLean SD Northrup H Scaglia F Strathearn L Trapane P Kang SH Patel A Cheung SW Hastings PJ Stankiewicz P Lupski JR Chromosome catastrophes involve replication mechanisms generating complex genomic rearrangements. Cell 2011 146 889 903 10.1016/j.cell.2011.07.042 21925314 PMC3242451

[B25] Quinlan AR Hall IM Characterizing complex structural variation in germline and somatic genomes. Trends Genet 2011 28 43 53 22094265 10.1016/j.tig.2011.10.002 PMC3249479

[B26] Conrad DF Bird C Blackburne B Lindsay S Mamanova L Lee C Turner DJ Hurles ME Mutation spectrum revealed by breakpoint sequencing of human germline CNVs. Nat Genet 2010 42 385 391 10.1038/ng.564 20364136 PMC3428939

[B27] Stephens PJ Greenman CD Fu B Yang F Bignell GR Mudie LJ Pleasance ED Lau KW Beare D Stebbings LA McLaren S Lin ML McBride DJ Varela I Nik-Zainal S Leroy C Jia M Menzies A Butler AP Teague JW Quail MA Burton J Swerdlow H Carter NP Morsberger LA Iacobuzio-Donahue C Follows GA Green AR Flanagan AM Stratton MR Massive genomic rearrangement acquired in a single catastrophic event during cancer development. Cell 2011 144 27 40 10.1016/j.cell.2010.11.055 21215367 PMC3065307

[B28] Berger MF Lawrence MS Demichelis F Drier Y Cibulskis K Sivachenko AY Sboner A Esgueva R Pflueger D Sougnez C Onofrio R Carter SL Park K Habegger L Ambrogio L Fennell T Parkin M Saksena G Voet D Ramos AH Pugh TJ Wilkinson J Fisher S Winckler W Mahan S Ardlie K Baldwin J Simons JW Kitabayashi N MacDonald TY The genomic complexity of primary human prostate cancer. Nature 2011 470 214 220 10.1038/nature09744 21307934 PMC3075885

[B29] Quinlan AR Clark RA Sokolova S Leibowitz ML Zhang Y Hurles ME Mell JC Hall IM Genome-wide mapping and assembly of structural variant breakpoints in the mouse genome. Genome Res 2010 20 623 635 10.1101/gr.102970.109 20308636 PMC2860164

[B30] Kidd JM Graves T Newman TL Fulton R Hayden HS Malig M Kallicki J Kaul R Wilson RK Eichler EE A human genome structural variation sequencing resource reveals insights into mutational mechanisms. Cell 2010 143 837 847 10.1016/j.cell.2010.10.027 21111241 PMC3026629

[B31] Keane TM Goodstadt L Danecek P White MA Wong K Yalcin B Heger A Agam A Slater G Goodson M Furlotte NA Eskin E Nellaker C Whitley H Cleak J Janowitz D Hernandez-Pliego P Edwards A Belgard TG Oliver PL McIntyre RE Bhomra A Nicod J Gan X Yuan W van der Weyden L Steward CA Bala S Stalker J Mott R Mouse genomic variation and its effect on phenotypes and gene regulation. Nature 2011 477 289 294 10.1038/nature10413 21921910 PMC3276836

[B32] Yalcin B Wong K Agam A Goodson M Keane TM Gan X Nellaker C Goodstadt L Nicod J Bhomra A Hernandez-Pliego P Whitley H Cleak J Dutton R Janowitz D Mott R Adams DJ Flint J Sequence-based characterization of structural variation in the mouse genome. Nature 2011 477 326 329 10.1038/nature10432 21921916 PMC3428933

[B33] Talbot CJ Nicod A Cherny SS Fulker DW Collins AC Flint J High-resolution mapping of quantitative trait loci in outbred mice. Nat Genet 1999 21 305 308 10.1038/6825 10080185

[B34] Valdar W Solberg LC Gauguier D Burnett S Klenerman P Cookson WO Taylor MS Rawlins JN Mott R Flint J Genome-wide genetic association of complex traits in heterogeneous stock mice. Nat Genet 2006 38 879 887 10.1038/ng1840 16832355

[B35] Deem A Keszthelyi A Blackgrove T Vayl A Coffey B Mathur R Chabes A Malkova A Break-induced replication is highly inaccurate. PLoS Biol 2011 9 e1000594 10.1371/journal.pbio.1000594 21347245 PMC3039667

[B36] Morrish TA Gilbert N Myers JS Vincent BJ Stamato TD Taccioli GE Batzer MA Moran JV DNA repair mediated by endonuclease-independent LINE-1 retrotransposition. Nat Genet 2002 31 159 165 10.1038/ng898 12006980

[B37] Zhang F Khajavi M Connolly AM Towne CF Batish SD Lupski JR The DNA replication FoSTeS/MMBIR mechanism can generate genomic, genic and exonic complex rearrangements in humans. Nat Genet 2009 41 849 853 10.1038/ng.399 19543269 PMC4461229

[B38] Manske HM Kwiatkowski DP LookSeq: a browser-based viewer for deep sequencing data. Genome Res 2009 19 2125 2132 10.1101/gr.093443.109 19679872 PMC2775587

[B39] Rozen S Skaletsky H Primer3 on the WWW for general users and for biologist programmers. Methods Mol Biol 2000 132 365 386 10547847 10.1385/1-59259-192-2:365

[B40] Yalcin B Willis-Owen SA Fullerton J Meesaq A Deacon RM Rawlins JN Copley RR Morris AP Flint J Mott R Genetic dissection of a behavioral quantitative trait locus shows that Rgs2 modulates anxiety in mice. Nat Genet 2004 36 1197 1202 10.1038/ng1450 15489855

[B41] Ewing B Hillier L Wendl MC Green P Base-calling of automated sequencer traces using phred. I. Accuracy assessment. Genome Res 1998 8 175 185 9521921 10.1101/gr.8.3.175

[B42] Gordon D Abajian C Green P Consed: a graphical tool for sequence finishing. Genome Res 1998 8 195 202 9521923 10.1101/gr.8.3.195

[B43] Wong K Keane TM Stalker J Adams DJ Enhanced structural variant and breakpoint detection using SVMerge by integration of multiple detection methods and local assembly. Genome Biol 2010 11 R128 10.1186/gb-2010-11-12-r128 21194472 PMC3046488

[B44] Database of Genomic Variants archive. http://www.ebi.ac.uk/dgva/

[B45] Smit AFAHR Green P RepeatMasker. http://www.repeatmasker.org/

