# Supplementary material for: The fine-scale architecture of structural variants in 17 mouse genomes
Source: Genome Biol. 2012 Mar 20;13(3):R18. doi: 10.1186/gb-2012-13-3-r18 (PMC3439969; doi:10.1186/gb-2012-13-3-r18)
Supplement: Additional file 1 — 21 PEM patterns. We found 11 'high-confidence' patterns and 10 'questionable' patterns. For each PEM, we provide PEM details, illustration using LookSeq [38] and PCR results. We show paired-end reads (black arrows) and how they map to the reference genome (dashed grey lines). Green arrows represent primer pairs. PCR was carried out across the founder strains of the HS [33]. We used HyperladderII as size marker. [file gb-2012-13-3-r18-S1.PDF]

Additional file 1: 21 PEM patterns

High Confidence PEM patterns | Example in Look Seq | PCR validation

H1

Deletion of 390 bp [11110101]
chr1:7,904,302-7,904,691 bp

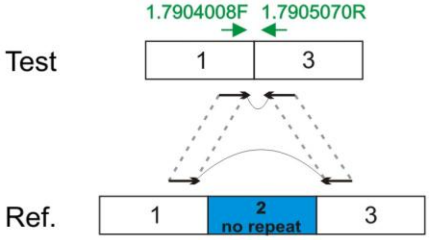

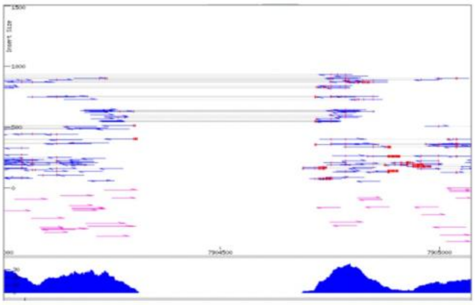

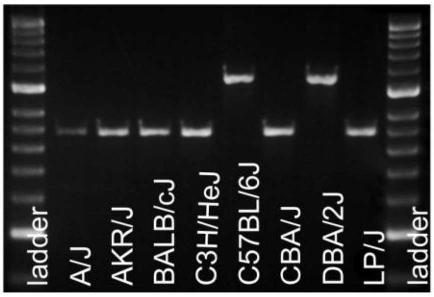

H2

Deletion of 656 bp [11110111]
chr8:31,595,128-31,595,783 bp

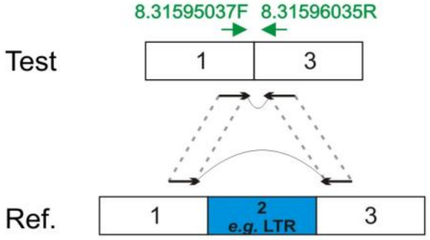

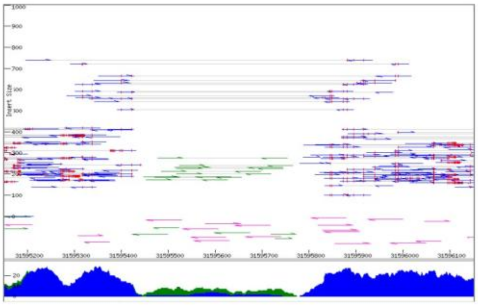

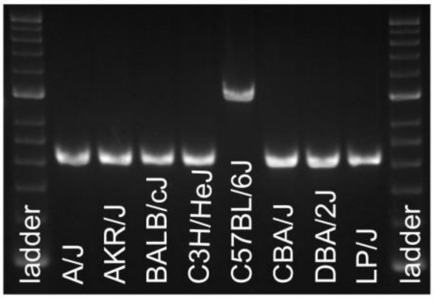

H3

del-384bp_nml-273_del-2414 [10010111]
1st del - chr15:90,971,314-90,971,697 bp
2nd del - chr15:90,971,971-90,974,384 bp

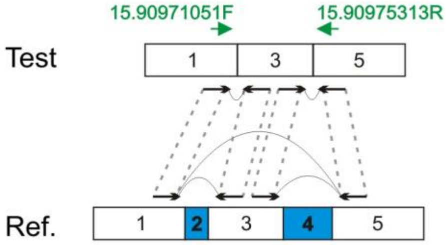

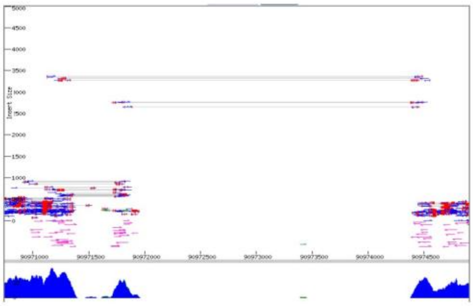

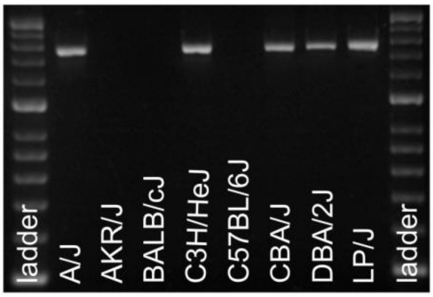

H4

Inversion of 1643 bp [00010111]
chr7:92,953,110-92,954,752 bp

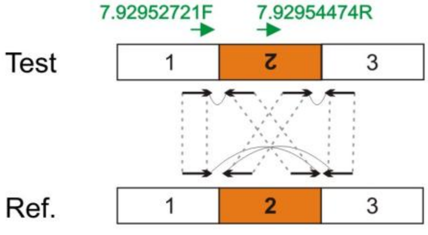

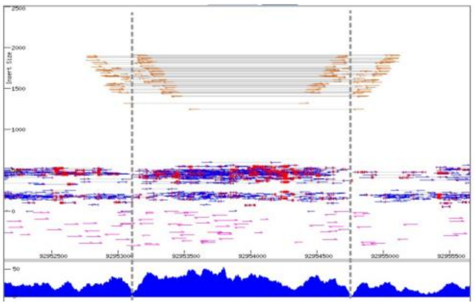

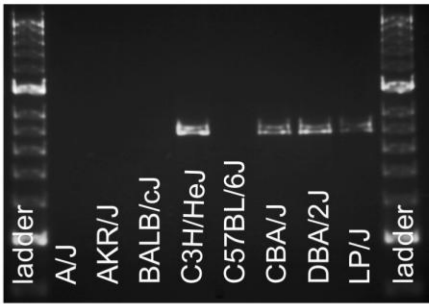

H5

del-71bp_inv-325_del-645 [11110000]
1st del - chr5:148,925,178-148,925,248 bp
inv - ch5:148,925,249-148,925,573 bp
2nd del - chr5:148,925,574-148,926,218 bp

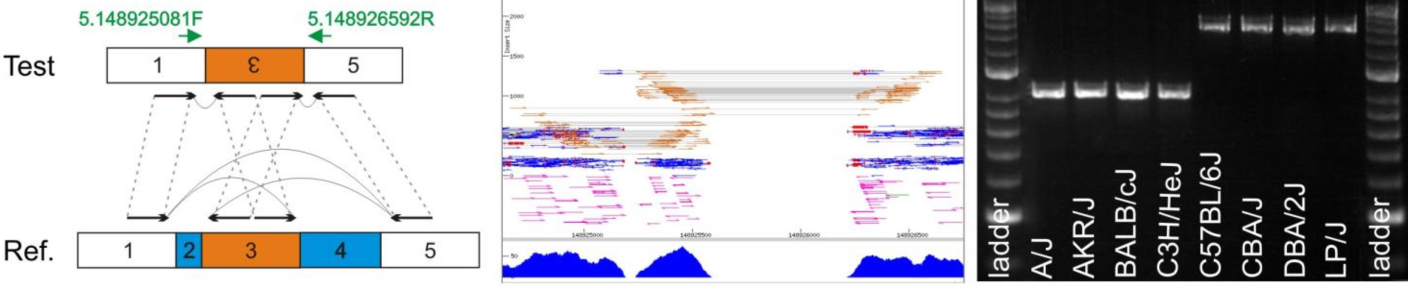

H6

Insertion of 391 bp [11110111]
chr8:32,237,946-32,237,948

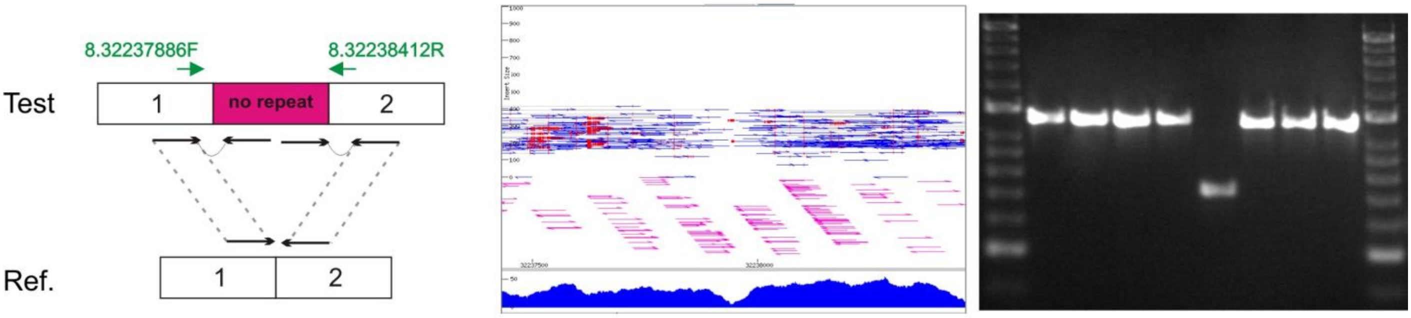

H7

Insertion of ~200 bp [00100010]
chr1:162,157,709-162,157,710

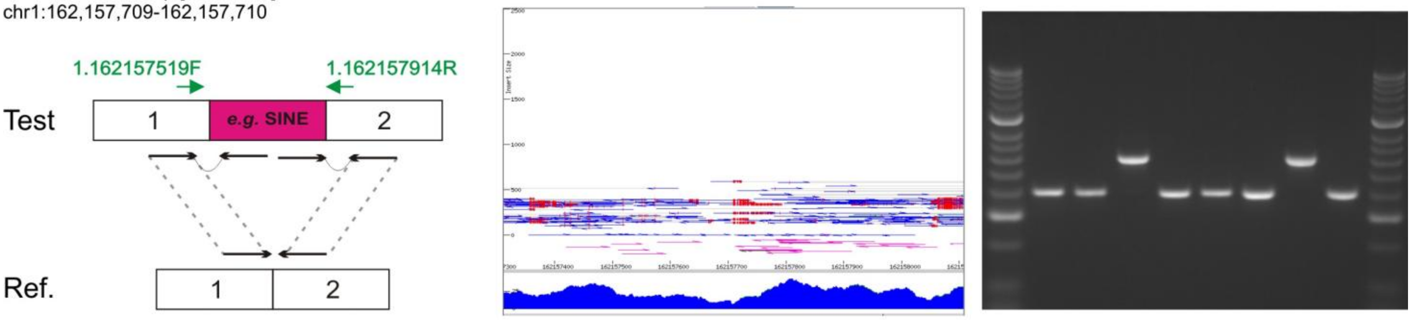

H8

Tandem duplication of 2181 bp [00010110]
chr19:59,423,833-59,425,976 bp

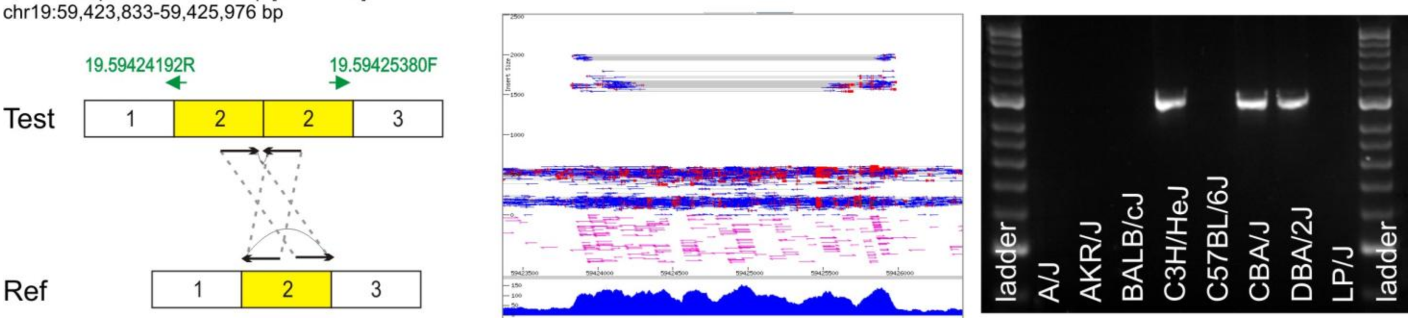

H9

Inverted tandem gain of ~128,6731 bp [01000000]
chr2:46,183,800-46,312,472 bp

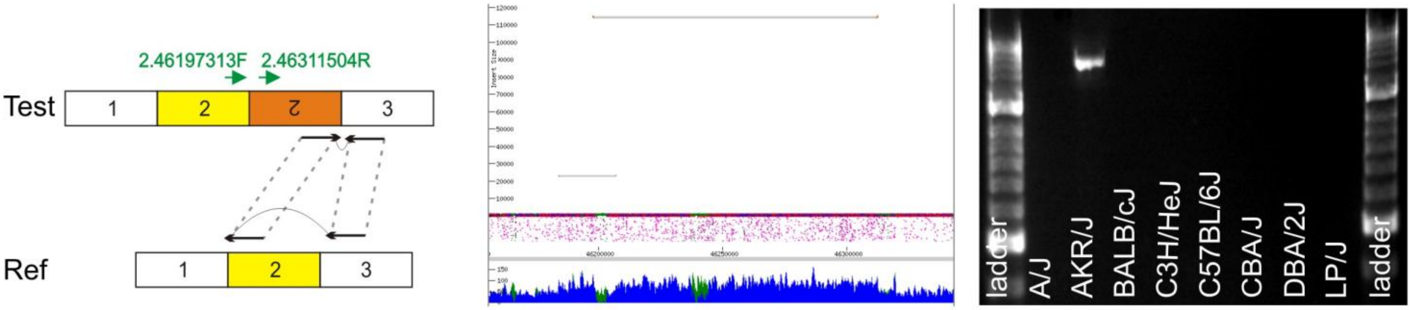

H10

Tandem gain of ~51681 bp with ins ~700bp [00100000]
chr2:172,253,836-172,305,516 bp

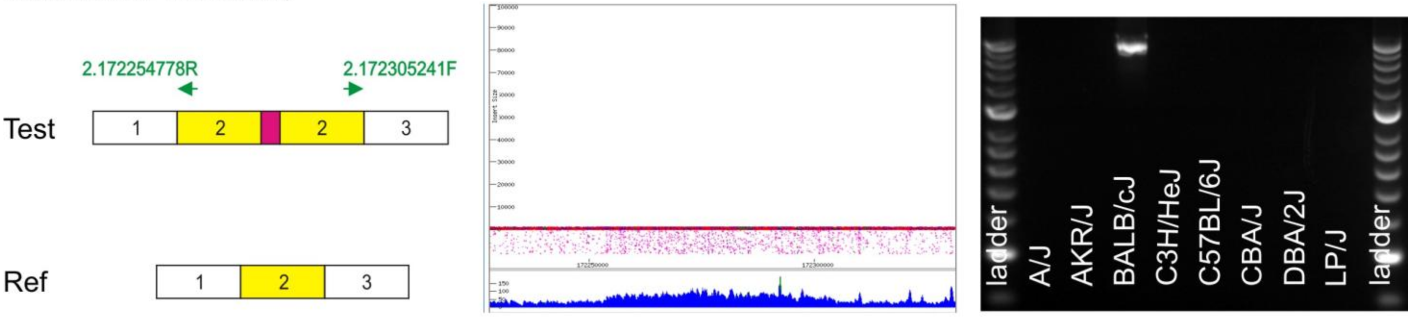

H11

Deletion in gain of ~1854 bp [00000010]
chr1:90,125,368-90,127,221 bp

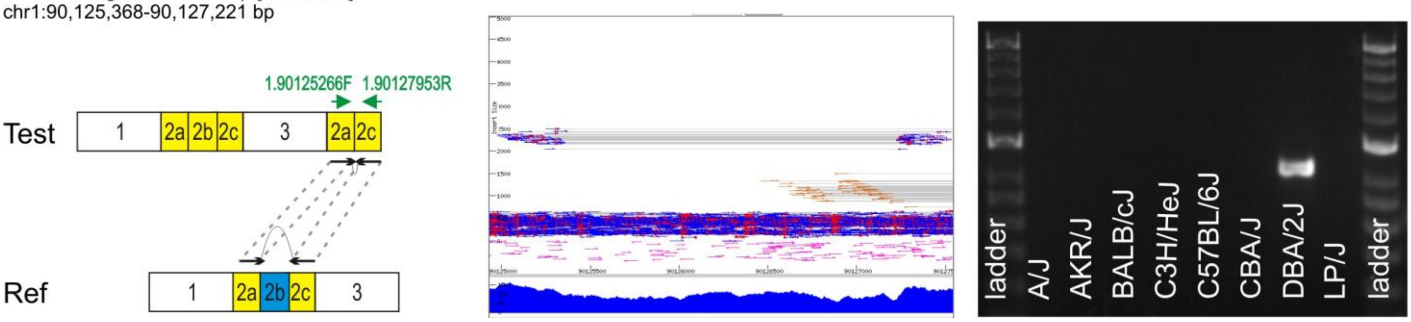

Questionable PEM patterns Example in LookSeq

PCR validation

Q1

Deletion of 185 bp [01110111]
chr19:57,325,727-57,325,911 bp

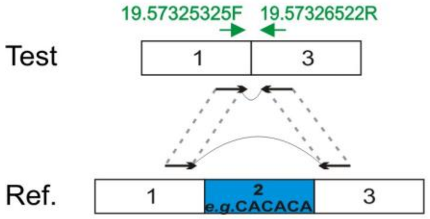

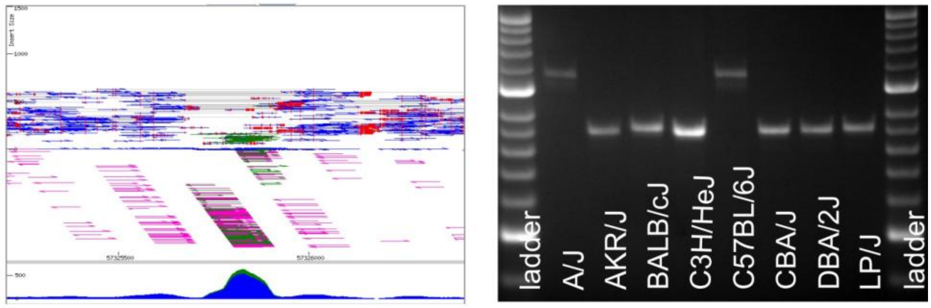

Q2

Deletion of ~ 836 bp with ins ~ 1200 bp [10100011]
chr19:48,061,057-48,061,892 bp

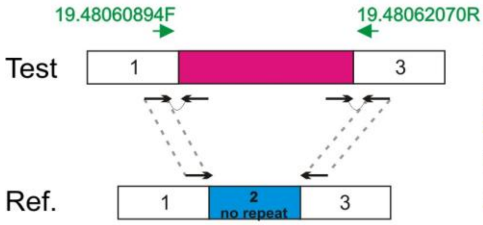

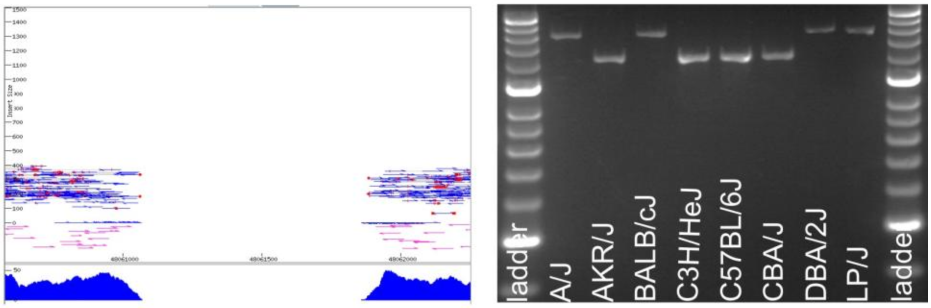

Q3

Deletion of ~ 163 bp with ins ~250 bp [10100000]
chr19:34,767,448-34,767,610 bp

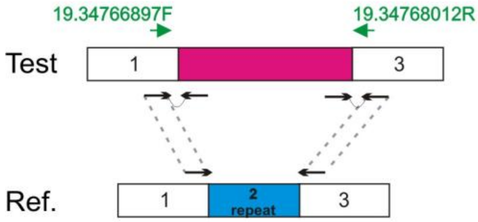

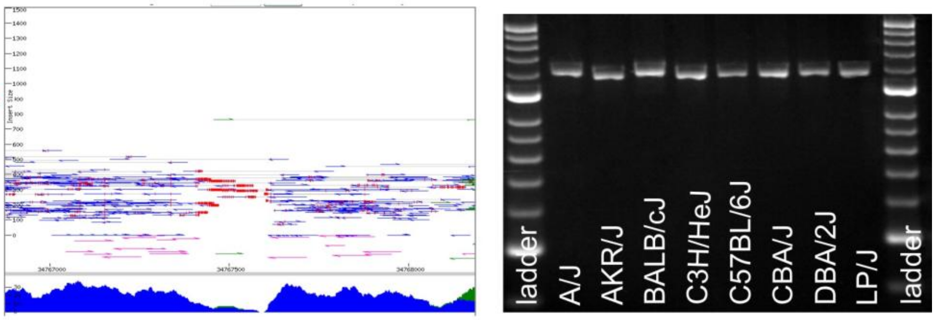

Q4

Deletion of 76444 bp [10100001]
chr16:36,244,199-36,320,642 bp

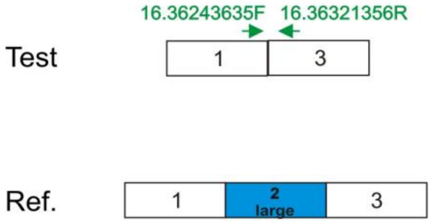

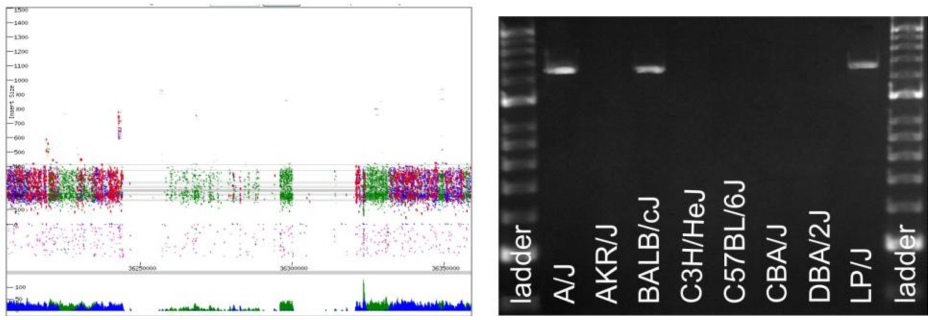

Q5

False deletion, instead insertion of 97 bp [11100001]
chr19:41,004,153-41,004,751 bp

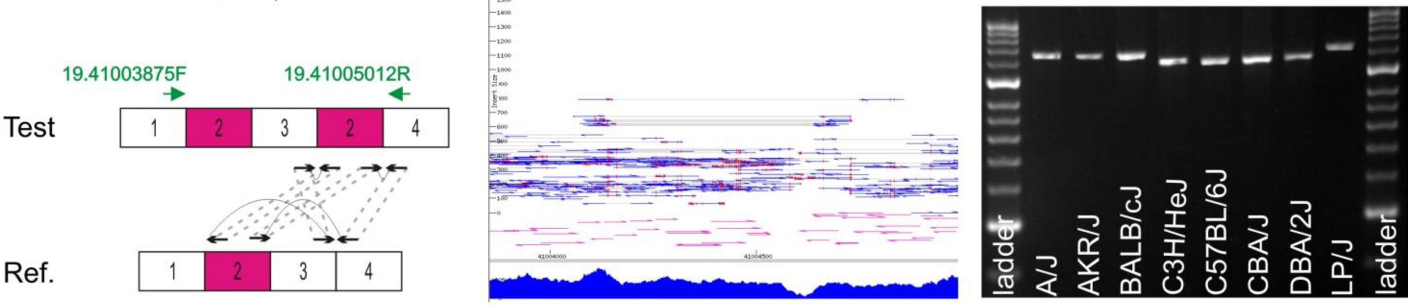

Q6

False deletion
chr19:15,980,612-15,981,451 bp

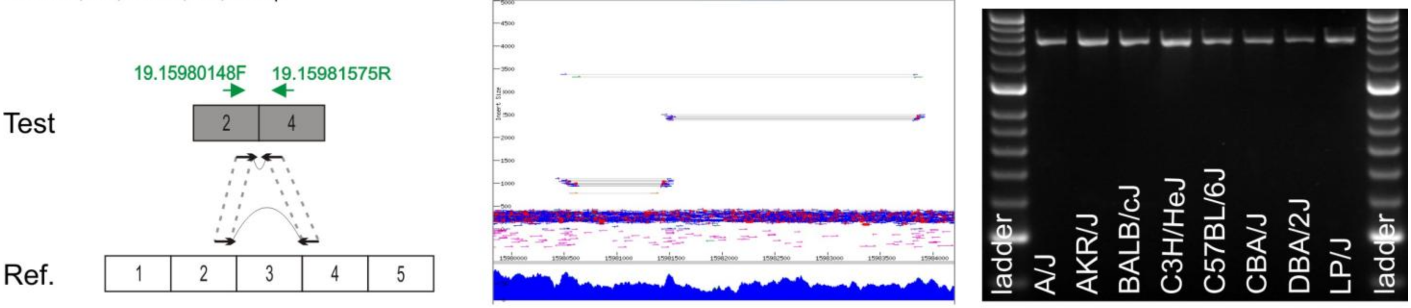

Q7

Deletion of variable size
chr19:44,194,931-44,195,258 bp

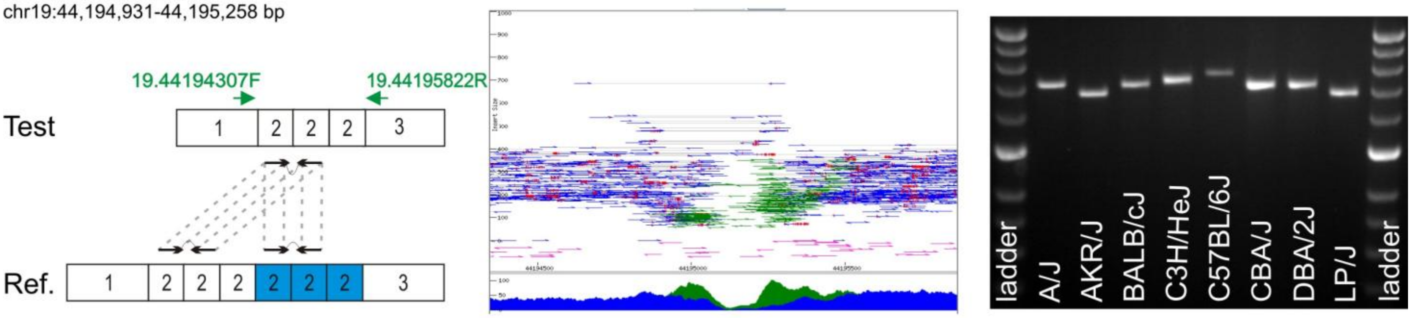

Q8

Inversion of ~ 548 bp with ins of ~400 bp [00110110]
chr8:77,137,213-77,137,760 bp

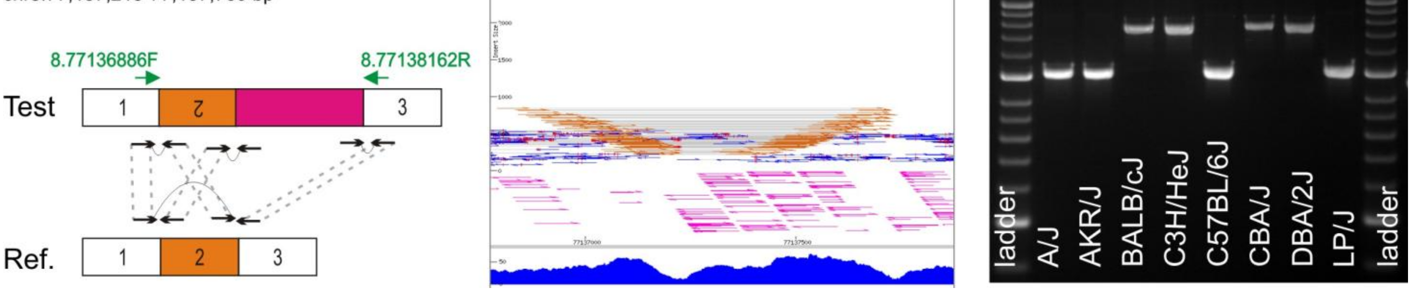

Q9

Inverted linked insertion of 68 bp [10110101]
chr3:127,740,121-127,740,698 bp

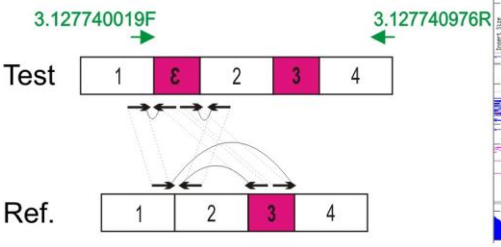

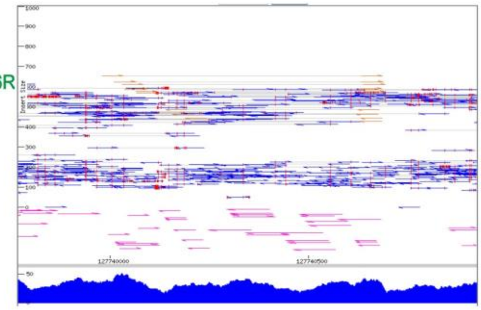

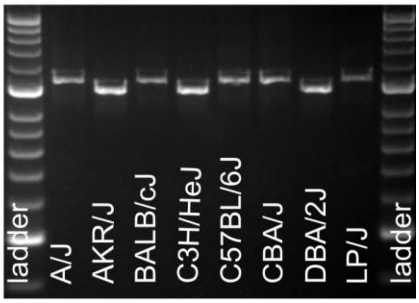

Q10

False inversion of about 7598 bp [11110111]
chr18:39,481,367-39,488,964 bp

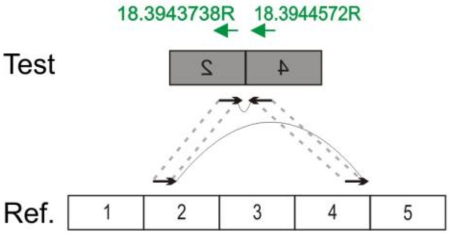

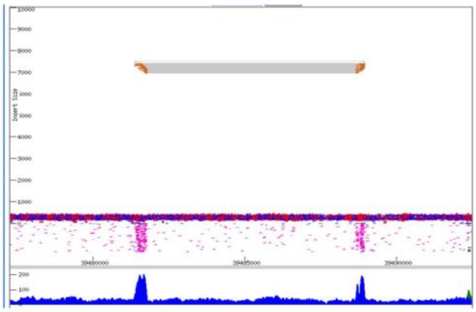

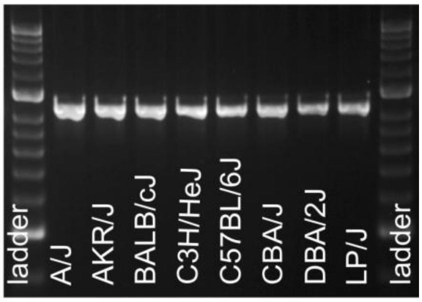

Pattern relative to the reference: Del Ins Inv Dup pseudogene
