# Supplementary figures and images for: The fine-scale architecture of structural variants in 17 mouse genomes
Source: Genome Biol. 2012 Mar 20;13(3):R18. doi: 10.1186/gb-2012-13-3-r18 (PMC3439969; doi:10.1186/gb-2012-13-3-r18)

### Additional file 3: Distribution of manual SV calls along chromosome 19

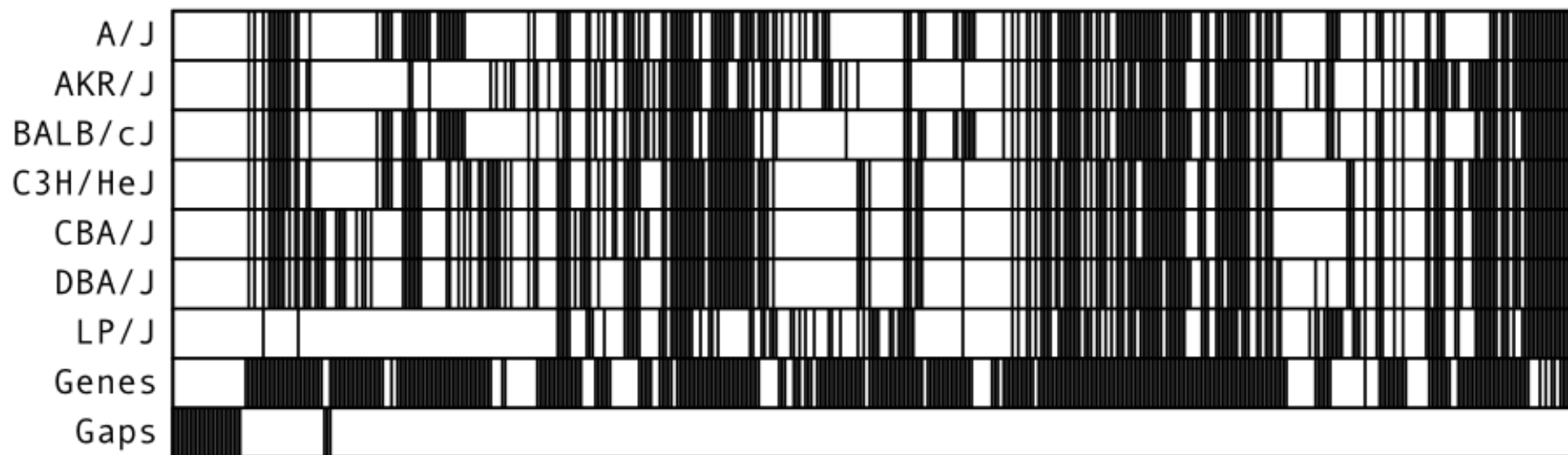

Supplement: Additional file 3 — Distribution of manual SV calls along chromosome 19. The top horizontal tracks show the chromosomal distribution of manually identified structural variants (deletions, inversions and duplications) for specific mouse strains (A/J, AKR/J, BALB/cJ, C3H/HeJ, CBA/J, DBA/2J and LP/J). The bottom two tracks represent genes (Ensembl 65) and gaps on chromosome 19. [file gb-2012-13-3-r18-S3.PDF]

Additional file 5: Primer design strategy

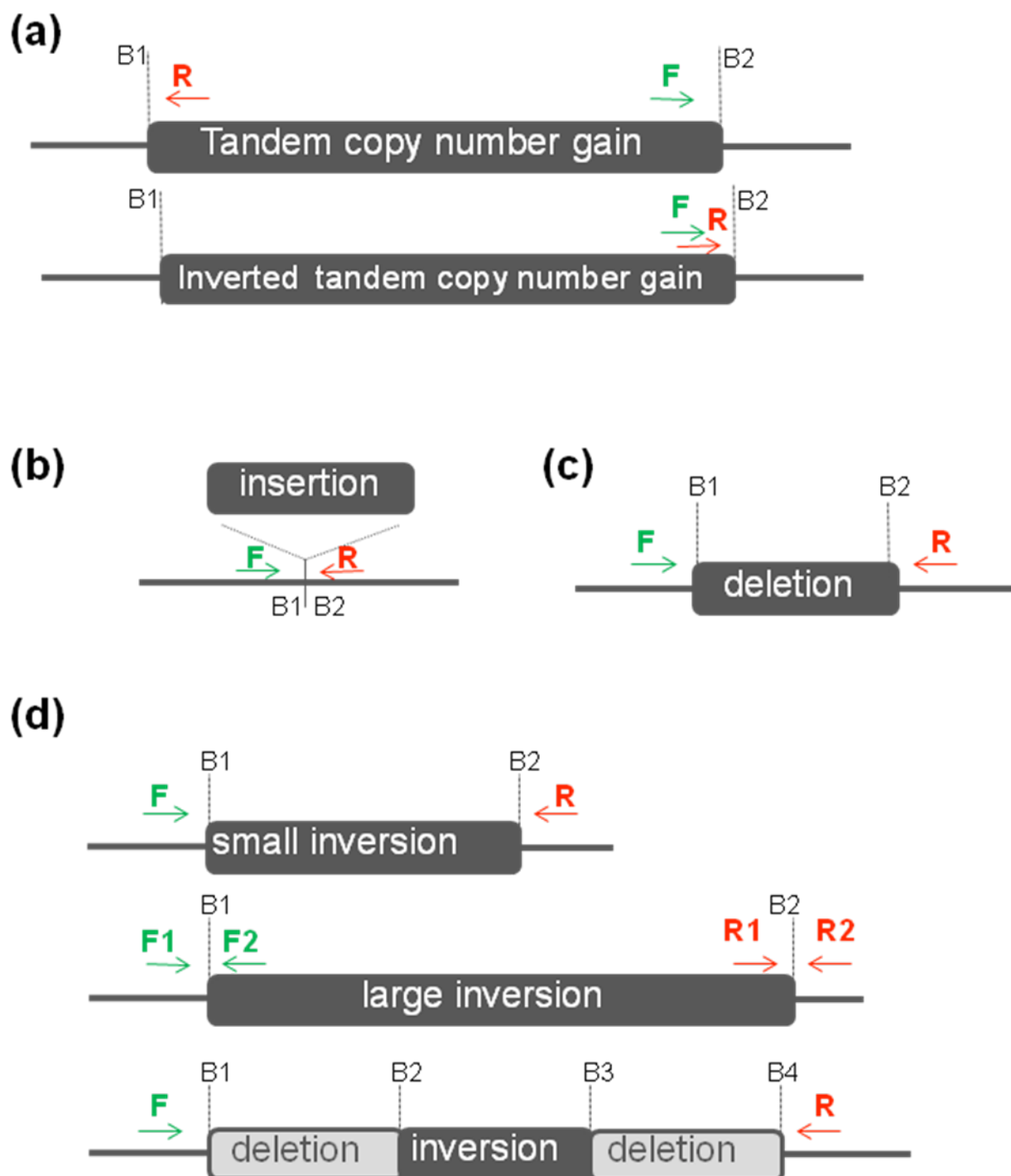

Supplement: Additional file 5 — Primer design strategy. We applied a primer design strategy depending on type and length of the SV. Forward primer is in green and reverse primer in red. SV sites were repeat masked prior to primer design, using RepeatMasker [45]. Breakpoints were initially predicted using LookSeq [38]. Primer design is illustrated for: (a) tandem duplication, (b) insertion, (c) deletion and (d) inversion. [file gb-2012-13-3-r18-S5.PDF]
